# The More and Less Study: a randomized controlled trial testing different approaches to treat obesity in preschoolers

**DOI:** 10.1186/s12889-015-1912-1

**Published:** 2015-08-01

**Authors:** Anna Ek, Kathryn Lewis Chamberlain, Jan Ejderhamn, Philip A. Fisher, Claude Marcus, Patricia Chamberlain, Paulina Nowicka

**Affiliations:** 1 grid.4714.60000000419370626Division of Pediatrics, B62, Department of Clinical Science, Intervention and Technology, Karolinska Institutet, 141 86 Stockholm, Sweden; 2 grid.410354.70000000102449440Oregon Social Learning Center, Eugene, OR USA; 3 grid.24381.3c0000000092415705Astrid Lindgren Children’s Hospital, Karolinska University Hospital, Stockholm, Sweden; 4 grid.170202.60000000419368008University of Oregon, Eugene, OR USA

**Keywords:** Children, Eating behaviors, Feeding practices, Lifestyle, Obesity, Parenting, Preschoolers, Self-efficacy, Socioeconomic status, Treatment

## Abstract

**Background:**

While obesity has been shown to be difficult to treat in school aged children and in adolescence, promising results have been detected for children who started treatment in early childhood. Yet knowledge on the effectiveness of structured early childhood obesity treatment programs is limited, preventing the widespread implementation of such programs. The main objective of this study is to evaluate the effectiveness of early treatment of childhood obesity with respect to treatment focus (parenting practices or lifestyle), length and intensity. The study will also examine the influence of gender, age, parental weight status, parenting practices, child behavior as well as parents’ socioeconomic status and child and parental psychosocial health on children’s weight status.

**Methods/design:**

This is a parallel open label randomized controlled trial assessing two different behavioral treatment approaches offered in three conditions to families with children aged 4–6 years in Stockholm County, Sweden. Children (n = 180) identified as obese will be referred from primary child health care, school health care, and from outpatient pediatric clinics, and randomized to: 1) a standard treatment with focus on lifestyle, provided within the current healthcare system (n = 90); 2) a 10-session, 1.5 h/week group treatment with focus on parenting (n = 45); or 3) the same group treatment as 2) with additional follow-up sessions (n = 45). The primary study outcome is change in children’s body mass index standard deviation score (BMI SDS) one year post-baseline. Secondary outcomes include changes in children’s waist circumference, metabolic health, lifestyle patterns (Food Frequency Questionnaire), obesity-related child behaviors (Child Eating Behavior Questionnaire and Lifestyle Behavior Checklist, Problem Scale), parents’ general and feeding parenting practices (Communicating with Children and Child Feeding Questionnaire) and lifestyle-specific self-efficacy (Lifestyle Behavior Checklist, Confidence Scale), family functioning (Family Assessment Device), child and parental psychosocial health (Child Behavior Checklist and Beck’s Depression Inventory II).

**Discussion:**

This study will facilitate a close examination of key components of treatment for obesity during early childhood and mechanisms of change. Results from this study will lead to better healthcare options for obesity treatment during early childhood and ultimately to the prevention of obesity later in life.

**Trial registration:**

ClinicalTrials.gov NCT01792531 Registered February 14, 2013.

## Introduction

While obesity has been shown to be difficult to treat in adults, adolescents and school-aged children, promising results have been seen for preschoolers. Yet knowledge on the effectiveness of structured childhood treatment programs for obesity early in life is still very limited, preventing the widespread implementation of such programs. The purpose of this paper is to present a study protocol for the randomized controlled trial, the More and Less Study (ML). The overarching aim of ML is to evaluate the effectiveness of early treatment of childhood obesity by evaluating two different treatment approaches offered in three conditions to families with children aged 4–6 years (n = 180) with obesity. The study is organized and performed within the healthcare system in Stockholm County, Sweden and participants are followed one year post-baseline.

## Background

### Early treatment

Although unprecedented high levels of obesity in children have been observed and discussed for more than a decade [1, 2], the effectiveness of managing childhood obesity in health care settings is not well-known. Reports from quality measurement organizations such as the Swedish Council on Technology Assessment in Health Care [3] and the Cochrane Collaboration [4, 5] conclude that the evidence for effective interventions in early childhood (≤5 years) is particularly limited. However, results from cohort studies indicate that early treatment can be more successful than treatment later in childhood. The first evidence for the efficacy of early childhood intervention was found in a study reporting long-term results from a cohort of over 600 children treated for obesity in Germany [6]. In this study, children in the youngest age group (4–7 years) clearly demonstrated the highest sustained decreases in body mass index standard deviation score (BMI SDS) at 5 years follow up after completing lifestyle treatment in comparison with children in older groups (8–10, 11–12 and 13–16 years) [7]. The findings were confirmed by researchers from the Karolinska Institutet (KI), demonstrating that if children with obesity were treated at younger ages (6–9 vs. 10–13 and 14–16 years) the results were significantly better 3 years after the treatment was initiated [8].

#### Length and intensity of treatment

There is no scientific agreement about the appropriate length or intensity of treatment in pediatric obesity. While obesity treatment in health care settings is usually low-intensive and spans many years, many classical structured obesity programs developed for children and adolescents are time-limited, usually lasting 3–4 months [4, 7, 9–12]. Brief interventions that build on a family’s existing resources can be equally effective in triggering successful and sustainable lifestyle modifications in comparison to more intensive efforts [9, 13, 14]. Braet and colleagues suggest that for a majority of children and adolescents a basic treatment proved to be enough [15]. Alternatively, others claim that continuous care is necessary due to the chronic nature of obesity [8, 16, 17]; thus, it would be unethical not to offer long-term treatment [18].

### Importance of parenting

The success of early interventions might be explained by allocating more attention to the role of parents in creating a healthy lifestyle for younger children [19, 20]. Parents ask for tools to manage their children’s problematic behavior (e.g., food fussiness especially with regard to vegetables, lack of satiety, emotional overeating and excessive screen time) [21]. Research indicates that parents of obese children report a higher frequency of these behaviors than parents with normal weight children [22] and also rate their confidence as lower in handling the problematic behaviors [23, 24].

Exclusive focus on parents in treatment has proved to be effective in improving child weight status [19, 20, 25–30]. Indirect focus on children might protect the child’s self-esteem [31], a concern commonly shared by parents [32]. Additionally, a parental focus has also shown to be a more cost-efficient and feasible approach [29, 33, 34]. Theoretically, programs targeting parents provide specific tools to parents over and above the standard recommendations around food and physical activity [35–38]. Tools can enhance general parenting and/or parenting practices in specific situations. General parenting is usually described as attitudes and beliefs that create an emotional climate and determine the behavioral expression between the child and the parent [39]. The four parenting styles commonly proposed in this context are: authoritative, authoritarian, permissive and neglectful [40, 41]. The parenting styles are built upon two dimensions of parental behavior; how responsive parents are to the child’s needs and how controlling they are of child behavior [40, 41]. An authoritative parenting style, characterized by being responsive to the child’s needs but able to set clear limits [40–42], has been associated with children eating healthier, having a higher physical activity level and a lower body mass index (BMI) [36, 43]. On the other hand, authoritarian, permissive and neglectful parenting styles have been associated to less healthy behaviors [36, 44]. However, the associations are affected by parenting practices in specific situations, such as feeding, as well as child and parental characteristics [36, 37, 39, 45–48]. Many researchers in childhood obesity have examined parental feeding practices, most often restriction [46, 49]. Restrictive and controlling feeding practices have been associated with a higher BMI in the child in many cross sectional studies [37, 50]. The relationships are less clear in longitudinal research [51, 52]. A better conceptualization of restriction and control is needed to understand the positive and negative effects on weight development [37, 45, 53, 54]. Parental monitoring has also been associated with healthy child behaviors and weight status, but the strength of associations is not as strong [49, 55–57], possibly due to parents awareness of the importance of such practices (social desirability) leading to high floor and ceiling effects in responses to items [58].

### General parenting as a treatment approach for preschool obesity

The predictive relationship between parents’ feeding practices and general parenting styles suggests a role for general parenting in childhood obesity interventions [59]. Parenting programs addressing general parenting aim to target child behavior indirectly by improving parents' knowledge, confidence and practices [60, 61]. Parents are made aware of specific behaviors that are effective when interacting with the child: encouragement, positive involvement, problem solving, emotional regulation, monitoring and limit setting strategies. These parenting practices have been linked to healthy child development and with a supportive family climate. On the other hand, behaviors such as inconsistent and over-reactive parenting practices have been shown to be maladaptive and are discouraged [60–63]. Few studies have included general parenting practices as part of a childhood obesity intervention [26, 28, 30, 64–66]; and only three studies have carefully assessed the changes in parenting practices during treatment [26, 28, 66] to establish the most important skills in shaping healthy lifestyles [35]. Moens and Braet conducted a pilot study targeting parents of school-aged children; the children’s weight status had improved 6 months post baseline; however, no changes were seen in general parenting practices which could be due to the small sample size [28]. In Australia, West and colleagues demonstrated promising results after a parenting program for parents with school-aged children; children’s weight status improved and parents increased their confidence in managing child obesity-related behaviors and used inconsistent or forceful parenting practices less frequently [26]. The same program was evaluated in the Netherlands but showed no long-term effects after twelve months [66]. Given the limitations of previous studies focusing on general parenting practices as a treatment approach for obesity and the lack of data for preschool aged children, there is a need for a new parenting focused program.

### Conceptual influences

Bandura’s Social Learning Theory [67] and Patterson’s Social Interaction Learning Theory [68, 69] state that children’s optimal development is promoted by active family involvement. Family members’ behavior modeling and effective interaction are therefore essential and result in a coherent and mutual understanding about responsibilities and role sharing. Equally important is the Ecological System Theory, developed by Bronfenbrenner [70], which further explains how children’s development is affected on many levels, not only by the microlevel contexts of their immediate environments (family, school, etc.). Child development is also influenced by mesolevel processes through which the children’s family, school, and other micro-environments interact, along with exolevel (community) and macrolevel (culture) contexts that do not involve the children directly [70]. Indeed, the cascading effects of Social Learning Theory based parenting programs, such as lower parental depression and improved families environment [71–73], demonstrate the direct and indirect contexts of child development.

#### Program development

One of the most effective parenting programs is KEEP (Keeping Foster and Kin Parents Supported and Trained). A major principle of KEEP, which is centered on Patterson’s Social Interaction Learning Theory, is that parents can serve as key agents of change for children. This is accomplished by strengthening caregiver confidence and skills so they can change their child’s behaviors, teaching effective parent management strategies, and providing them with support. Five dimensions of positive parenting form the cornerstone of KEEP. *Encouragement* involves the use of scaffolding (i.e., breaking complex behaviors into achievable steps and encouraging approximations toward the goal) and positive reinforcement to teach new behaviors. *Limit setting* involves using clear instructions and being consequent to discourage negative behaviors. *Monitoring* involves parental tracking of child whereabouts, activities, and behaviors and the provision of appropriate adult supervision. *Problem solving* involves setting goals, developing strategies to achieve goals, committing to the decision, trying it out, and making relevant adjustments. *Positive involvement* involves how parents show love and interest to their child [60, 74]. The intervention moves in a step-by-step fashion, with parents learning one positive parenting skill before adding new skills to their parenting tool box [60, 74]. Role play is used extensively as a teaching tool to help parents to understand situations from differing perspectives. Role play also provides practice with a group leader to ensure the use of skills from the group sessions to the home.

KEEP was developed by Dr. Patricia Chamberlain and colleagues at the Oregon Social Learning Center (OSLC), a family research institute in the USA. As the intervention has been used previously with foster families in Sweden, a natural step was to use KEEP as a foundation for a childhood obesity parent program in Sweden. Modifications of the program are described under “Methods”.

**Fig. 1:**
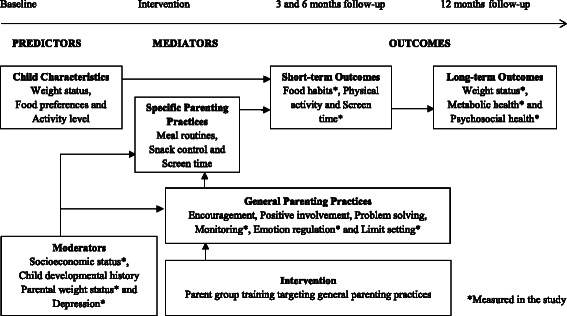
Conceptual model of the relationships between parenting and child weight status

According to our conceptual model (see Fig. 1), changes in general parenting (e.g., encouragement, limit setting) will modify specific parenting practices (e.g., meal and screen time situations and routines) that will consequently influence the child’s home environment and result in improved child obesity outcomes such as BMI SDS. An important supporting component in the new program is the *regulation of emotional expression* within each of the five positive parenting practices inspired by the Parent Management Training Oregon Model (PMTO) [71, 75].

### Mechanisms for treatment effect

Because parental behavior is affected by child characteristics and behaviors [39, 76, 77] the pathways for how these associations predict, mediate or moderate treatment results need to be assessed. Research on which dimensions of general child behaviors (internalizing and externalizing) that children with obesity are most vulnerable to is still inconclusive [78]. Identifying a child behavior profile before treatment start was proposed in a Dutch study as a way to direct families to a more appropriate obesity treatment [78]. In the same study the authors stressed that the weight status alone was not an indicator for psychological health [78]. Children’s eating behavior (based on research on appetitive traits) is also a behavioral risk factor for obesity that we will examine [79]. Children with overweight and obesity tend to be more responsive to food and enjoy food more, have a higher degree of emotional eating, lower response to internal satiety cues and are less fussy when it comes to food compared to healthy weight peers [80].

Among moderators, parental depression may have especially profound consequences on treatment outcomes. Parental depression has previously shown moderating effects on parenting style as well as for specific practices and child obesity; depressed mothers were more permissive and used less controlling feeding practices [52, 81].

Family’s socioeconomic status (SES) measured as parental education is usually assessed in interventions and has been linked to treatment outcomes [82–85]. Other components of SES such as income, social support (functional and structural), family and household structure and economic stress have not yet been examined in detail; such examinations would lead to a better understanding of the influence of other factors beyond education. Parent’s foreign origin is another background factor of influence for a healthy lifestyle such as children’s dietary intake [86, 87].

### Aims and hypothesis

The overarching aim of ML is to evaluate two different treatment approaches for obesity in preschool aged children offered in three conditions. The specific aims are:

1. To determine the effectiveness of two obesity treatment approaches: 1) standard treatment (n = 90) and 2) parent group treatment (with and without booster sessions) (n = 90). The two approaches will be evaluated with respect to child weight status, BMI SDS (primary outcome). The secondary outcomes for the child will be: lifestyle patterns, child eating and obesity related behavior, metabolic and psychosocial health; for parents: family functioning, parental feeding and limit setting practices, confidence and depression.
2. To examine the influence of treatment length and intensity (as defined by group attendance and participation in booster sessions) for the two different parent group conditions; 1) 10 weekly 1.5 h sessions (n = 45) and 2) 10 weekly 1.5 h sessions plus booster sessions every 6 weeks during the remaining time of the study (n = 45).
3. To examine predictors, moderators and mediators of treatment effects. We will assess whether changes in general practices (limit setting and emotional regulation) and parental feeding practices (restriction, pressure to eat and monitoring) will mediate child obesity outcomes by examining all treatment groups. In addition, we will examine predictors and moderators of intervention effects (e.g., age, gender, socioeconomic status (SES), child eating behavior, child psychosocial health, parental confidence, weight status, and depression).

We hypothesize that the ML parent group treatment, which addresses obesity in preschool aged children, will be effective in improving both primary and secondary outcomes. We expect that core elements of the parent group program (i.e., encouragement, positive involvement, problem solving, emotional regulation, monitoring, and limit setting strategies) will have additional beneficial effects on child weight status by helping parents to support health-promoting routines and habits around food/beverage consumption and physical activity practices to reduce sedentary behaviors compared to standard treatment; see our conceptual model in Fig. 1. Further, we assume that additional booster sessions in the parent group treatment condition will significantly improve treatment outcomes showing that regular follow-up is necessary in the treatment of childhood obesity.

## Methods/Design

### Design of the study

This is a parallel open label randomized controlled trial comparing effects of two obesity treatment approaches for 4–6 year old children: standard treatment as offered in pediatric clinics in Stockholm County, Sweden, and a parent group treatment focusing on parenting practices supporting healthy lifestyle changes. The parent group treatment is offered in two conditions in order to assess the effects of treatment intensity and length. The trial commenced 2011 and will run until 2017.

### Ethical approval

The study was approved by the Regional Ethical Board in Stockholm (ID: 2011/1329-31/4) on 16^th^ of November 2011 with amendments (2012/1104-32; 2012/2005-32; 2013/486-32). The protocol for the study is registered with the clinical trials registry clinicaltrials.gov (ID: NCT01792531).

### Recruitment process

The study is occurring in Stockholm County, Sweden. Recruitment started in 2012 and is expected to last until 2016. As a first step, a study steering committee was developed consisting of key representatives from the child health care system in Stockholm County. The representatives identified crucial individuals in the primary and secondary health care centers to facilitate recruitment, provided ideas for optimal recruitment as well as for how the set up for the standard treatment could be designed. They also provided contacts for professionals working in primary child health care centers, secondary outpatient pediatric clinics and school health offices. All health care representatives have approved the study protocol. The next step for the research group was to provide further information about the study through individual visits, telephone calls, emails and regular mail contact, which will be upheld for the duration of the study.

Primary child health care nurses serve as our main recruitment source. In Sweden all parents of children 0–5 years old are offered yearly health check-up visits to the primary health care free of charge and 99.5 % of the population reach the minimum number of recommended visits [88–90]. In part, the high attendance can be explained by the close relationship that often develops between the family and the nurse during the infant’s first year [91]. During the visits the nurse measures the child’s weight and height and records the values into the weight, height and BMI charts. The child’s growth and development is then discussed with the parents [92]. We established the following protocol for our study: when the family comes for the routine health check-up at 4 and 5 years and obesity is detected, according to the international cut-off criteria recommended by Cole et al. [93], the nurse provides a short description of ML to the parents. The close relationship with the nurse can facilitate the often sensitive conversation. If the parents want to receive further information about the study and agree to be contacted by the research group, the health professionals send the family’s contact information and child weight and height charts to the research group. Parents can also contact the research group by phone or email themselves after the visit. Detailed study information and a letter of consent are then sent to the family. After a week the family is contacted by the researchers offering to answer questions that may have arisen. Parents who want to participate in the study are then asked to sign the letter of consent and send it back to the research group; a copy signed by a member of the research group is then sent back to the family.

Children can also be recruited during visits at outpatient pediatric clinics (secondary health care) to which children normally are referred if obesity is detected. The school health care system is also involved in the recruitment because of its health promoting role in primarily working with preventive and health supportive tools. On at least three occasions in school, routine health check-ups are provided; the first during the preparation year for 6-year-old children [94]. The visits include measures of weight and height [95].

The fourth source of recruitment is self-recruitment through ads in local papers and through community bulletin boards.

### Participants

We aim to include 180 families with children aged 4–6 years with obesity as defined by the age and gender specific international cut-offs for BMI [93]. Parents who agree to participate in the study sign an informed consent before study participation begins.

#### Inclusion and exclusion criteria

Eligible families are included according to the following criteria:

1. Child’s age between 4 and 6 years (i.e., up to but not including reaching their 7^th^ birthday before start of treatment); and
2. Child’s obesity according to international cut offs for BMI in children [93]. For practical reasons obesity was defined as BMI 19.2, independent of age and gender.
3. No chronic disease or developmental problem that is likely to influence child weight and height; and
4. Parental ability to understand and communicate in Swedish to fill out questionnaires and participate in treatment.

### The power calculation

The power calculation was based on data from Kleber et al. [7]. We hypothesize that standard treatment will be slightly less effective than the intensive treatment obtained in the German study (we assume -0.3 BMI SDS compared to -0.46). We expect a dropout rate of 21 % based on the Australian study by West and colleagues with similar design but older children [26]. We hypothesize that the parent group treatment will be twice as good as standard treatment with regard to the primary outcome BMI SDS (i.e., -0.6 one year post baseline). The calculation is based on pairwise/three group comparisons. Based on these assumptions we will require at least 75 children in each group (*p* < 0.05 and power 85 %) to be able to detect changes between two groups; standard treatment and parent group treatment (primary outcome).

#### Allocation

**Fig. 2:**
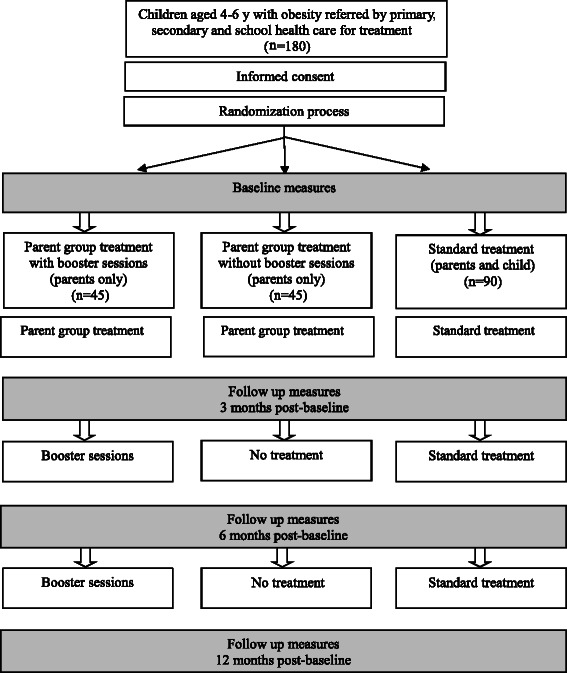
Study design

Eligible families are randomly assigned (2:1:1) to one of three treatment conditions (standard treatment, parent group treatment with booster sessions, parent group treatment without booster sessions) using an electronic randomization program with permuted blocks. Randomization sequence is maintained by the study statistician to assure concealment. Neither the parent group leaders nor the families are informed about which families are allocated to boosters until after the groups are finished in order to avoid differential treatment (Fig. 2).

### Treatment approaches and settings

#### Standard treatment

Children randomized to standard treatment will receive treatment as usual in an outpatient pediatric unit. The treatment will be provided by local pediatricians and nurses and will be based on lifestyle modifications, as recommended in the action plan for Stockholm County. Families will receive at least 4 treatment visits: the first is typically a pediatric evaluation with the physician and the subsequent visits are with the pediatric nurse. A dietician and physiotherapist will be consulted when a need arises. The visits center around lifestyle advice, such as how parents can help the child to increase physical activity and modify eating habits. In the beginning of the treatment reasons for weight gain and future risk factors are discussed. At the follow-ups parents are offered support both for the changes that they have achieved and receive advice on areas for future improvement. Because the treatments vary between pediatric units we have developed a special questionnaire including questions about the profession of the health care providers involved as well as the number and focus of visits.

#### Parent group treatment

In a close collaboration with the KEEP developers we translated and adapted the parent group manual outlining the content of each session and parent handouts. The standard 16-session program was shortened to a 10-session program, 1.5 h per week, for Swedish parents with preschoolers with obesity following cultural adaptation (especially for limit setting strategies). Furthermore, new parent material containing tailored advice on food and physical activity was developed.

Each session centers around a parenting practice that is presented, discussed and practiced. See Table 1 for a description of session content. Each session also includes a lifestyle component (regarding food and physical activity/sedentary behaviors) aimed to provide parents with knowledge and skills to support a healthy home environment. The sessions finish with a home practice assignment related to the focus of the day. Two group leaders (one group leader and one co-leader) with health care backgrounds facilitate the sessions that consist of 6–10 parents. Both parents are invited and child care is provided. All sessions are videotaped and analyzed with respect to program fidelity and program improvements [96].

**Table 1:** Content description of the parent group program

Session	
1.	Welcome and Introduction: Parents’ Key Roles
2.	Food and Play: When More, When Less?
3.	Cooperation
4.	Parents as Teachers
5.	Charts and Incentives
6.	Pre-teaching and Planning
7.	Limit Setting Strategies
8.	Power Struggles
9.	More Support – Less Stress
10.	Summary: Parenting and Balancing Food and Play

The initial training of the ML group leaders was conducted by the KEEP developers. The five-day experiential training included information on the program’s theory and practice in the delivery of group sessions. During the training each session of the ML program was thoroughly discussed and each trainee role practiced facilitating several key sessions while other trainees acted as parents. The training then continued through external supervision from the KEEP developer group; all parent group sessions were videotaped using a laptop with software that enables the recording to be uploaded to a secure website and were translated to English. The KEEP consultant viewed the recordings, rated them for fidelity, and identified areas for reinforcement and feedback. The recordings then were used in weekly consultation meetings (1 h each). Prior to the consultation, group leaders completed a session review form with questions about what went well and challenges experienced. They also completed weekly forms on parent attendance and engagement ratings. Each of these measures informed the consultation process. After completing three supervised groups the group leader can apply for certification as ML leader. After certification by the KEEP model developers, the group leaders receive bi-annual fidelity checks. After co-leading two groups, a co-leader person can apply for certification after additional supervision during one group as group leader.

Families allocated to booster sessions receive phone calls every four to six weeks for the remaining nine months of the study. During these 30 min calls, parents are encouraged to maintain lifestyle changes regarding food and physical activity/sedentary behaviors already made, as well as receive support for new challenges they are facing. Subsequent calls begin with a follow-up of what was discussed during the previous call. In the booster sessions the parents are also referred back to discussions held during the program and to the manual for additional support.

### Primary and secondary outcomes

The outcome measures are collected at baseline, 3-, 6- and 12-months post-baseline; see primary and secondary outcome measures in Table 2. The primary outcome measure is BMI SDS, derived from Swedish age and gender specific reference values [97]. Body mass index (BMI) (= weight in kilograms/height in meter^2^) will be calculated to be able to create weight status categories according to international cut-offs [93] to be able to examine changes during the study time.

**Table 2:** Socio-demographic characteristics and outcome measures collected at different time points

		Measure	Baseline	3 months	6 months	12 months
**Child**						
	Weight/height	Measured by health professionals	x	x	x	x
	Waist circumference		x	x	x	x
	Blood pressure		x	x	x	x
	Blood samples (glucose, HbA1c, insulin, CRP, kolesterol, LDL kolesterol, HDL kolesterol, triglyceriders, liver status, urate, TSH)		x			x
	Date of birth	Child background questionnaire	x			
	Country of birth		x			
	Sex		x			
	Health status		x			x
	Day care		x			x
	Visits to health care regarding child weight		x			x
	Sedentary behavior		x	x	x	x
	Food habits	Food frequency questionnaire	x	x	x	x
	Eating behavior	Child Eating Behavior Questionnaire	x	x	x	x
	Behavior	Child Behavior Checklist	x			x
**Parent**						
	Weight/height	Parent background questionnaire	x			x
	Date of birth		x			
	Country of birth		x			
	Sex		x			
	Education level		x			
	Health status/weight reducing operation		x			x
	Occupation status		x			x
	Income		x			x
	Family structure		x			x
	Social and economic support from network		x			x
	Perceived level of comfortable life		x			x
	Perceived child problem behavior and parental confidence	Lifestyle Behavior Checklist	x	x	x	x
	Feeding practices	Child Feeding Questionnaire	x	x	x	x
	Limit setting strategies	Communicating with children	x	x	x	x
	Family function	Family Assessment Device	x			x

The socio-demographic questionnaires were specifically designed for this study using items from established instruments. The questionnaire development was conducted by the research team in collaboration with family psychologists from the OSLC, USA, and with anthropologists at the University of Oxford, UK contributing expert knowledge of the socio-cultural importance for obesity development.

#### Anthropometrics

Child height is measured to the nearest 0.1 cm using a fixed stadiometer. Children are weighed to the nearest 0.1 kg wearing underwear. BMI is calculated based on weight and height. Waist circumference is measured to the nearest 0.1 cm at the midpoint between the lower rib and the iliac crest using a non-extensible tape. Systolic and diastolic blood pressure is measured using an automatic blood pressure monitor. Each measure is performed three times and mean values are then calculated. For all children anthropometrics are measured in a standardized manner by trained health care professionals with calibrated instruments.

#### Metabolic markers and blood pressure

Fasting blood samples are collected at baseline and at 12 months and taken at the family’s local primary health care facility. The metabolic markers examined are: P-glucose, B- HbA1c, P-cholesterol, P- LDL-cholesterol, P-HDL-cholesterol, P-triglycerides, P-ALAT, P-ASAT, P-urate, S-TSH, P-CRP and S-insulin. These metabolic markers are recommended for annual tests in pediatric obese patients.

#### Child food and physical activity habits

Dietary intake is assessed by a short version of an established food frequency questionnaire. To assess eating patterns, additional questions included: the child’s breakfast habits, who is responsible for the child’s food and physical activity and peer influence on the child’s food and physical activity. The questions regarding the child’s sedentary behavior focused on child screen time during weekdays and weekends.

The instruments used to measure child and parental behaviors are presented in Table 3 and briefly described below.

**Table 3:** Measures used in the study

Name of Instrument	Reference	Domains measured	Number of items	Brief description of measures:
Child Eating Behavior Questionnaire (CEBQ)	Wardle et. al. 2001		35	
		*Food Approach*		
		Food Responsiveness (FR)	5	The child’s general appetite.
		Enjoyment of Food (EF)	4	The child’s interest in food.
		Emotional Overeating (EOE)	4	If the child eats as a response to emotions.
		Desire to Drink (DD)	3	The child’s desire to drink
		*Food Avoidance*		
		Satiety Responsiveness (SR)	5	If the child gets full easily or not.
		Slowness in Eating (SE)	4	The child's speed of eating.
		Emotional Undereating (EUE)	4	If the child eats less as a response to emotions.
		Fussiness (FU)	6	The child eats a limited variety of food.
Lifestyle Behavior Checklist (LBC)	West and Sanders 2009 West et. al. 2010	Problem Scale/Confidence Scale	25	Parent’s perceptions of child obesity related problem behavior. Parent’s confidence in handling problematic behaviors.
		Overeating (OE)	7	If the child eats large potions or often asks for food.
		Misbehavior in relation to food (MB)	7	If the child throws tantrums about food or gets angry if not given food.
		Emotional correlates of being overweight (EMO)	5	If the child complains about e.g. peer problems, clothes being too small.
		Physical Activity (PA)	5	If the child is reluctant to physical activity and engages in sedentary behaviors.
Child Feeding Questionnaire (CFQ)	Birch et. al 2001			
		Perceived Responsibility (PR)	3	Parental perception of their responsibility for child feeding.
		Parent Perceived Weight (PPW)	4	Parental perception of their own weight status history.
		Perceived Child Weight (PCW)	6	Parental perception of child weight status history.
		Concern about child weight (CN)	3	Parental concern about the child’s risk of overweight.
		Monitoring (MN)	3	The extent to which parent’s oversee the child’s food intake.
		Restriction (RST)	8	The extent to which parents restrict the child’s access to food.
		Pressure to Eat (PE)	4	Parent’s tendency to pressure the child to eat more food.
Family Assessment Device (FAD)	Epstein et. al. 1983			
		Problem Solving	5	Ability to resolve problems in the family.
		Communication	6	Exchange of clear and direct verbal information.
		Roles	8	Division of responsibility for completing family tasks.
		Affective Responsiveness	6	Ability to respond with appropriate emotion
		Affective Involvement	7	Degree to which family members are involved and interested in one another.
		Behavior Control	9	Manner used to express and maintain standards of behavior.
		General Functioning	12	Overall function in the family.
Child Behavior Checklist (CBCL)	Achenbach and Rescorla 2000		99	
		*Internalizing Scale*		
		Emotionally Reactive	9	The child is easily disturbed, has mood swings etc.
		Anxious/Depressed	8	The child is overly sensitive, clings to parent or too independent, sad etc.
		Somatic Complaints	11	The child has aches, pain or vomits with no medical reason etc.
		Withdrawn	8	The child shows little interest in people or surroundings, doesn’t answer etc.
		Sleep Problems (not included in the Internalizing Scale)	7	The child doesn’t want to sleep alone, has nightmares, has little sleep etc.
		*Externalizing Scale*		
		Attention Problems	5	The child can’t concentrate or sit still, wanders away et.
		Aggressive behavior	19	The child is angry, defiant, disobedient, demanding, stubborn etc.
Beck’s Depression Inventory II (BDI-II)	Beck et. al 1988	Mood, Pessimism, Sense of Failure, Lack of Satisfaction, Guilt Feelings, Sense of Punishment, Self-dislike, Self-accusation, Suicidal Wishes, Crying, Irritability, Social Withdrawal, Indecisiveness, Distortion of Body Image, Work Inhibition, Sleep Disturbance, Fatigability, Loss of Appetite, Weight Loss, Somatic Preoccupation and Loss of Libido.	21	Symptoms and attitudes to assess intensity of depression.
Communicating with Children	(self-developed)		12	
		Limit Setting Strategies		Parents limit setting strategies (consequent or not)
		Emotional Regulation		Parents ability of emotional control

#### Child obesity related behavior

The Child Eating Behavior Questionnaire (CEBQ) includes 35 items on eating styles related to obesity risk, measured on eight factors [79]. The first four factors represent the dimension ‘food approach’ and the other four factors represent ‘food avoidance.’ Parents rate each behavior on a five-point Likert scale (never, rarely, sometimes, often, always; 1–5). The CEBQ has proved to have a good validity and high internal reliability in several studies [79, 98–103].

The Lifestyle Behavior Checklist includes 25 items divided on two scales: the Problem scale and the Confidence scale. On the Problem scale, parents rate to what extent an obesity related behavior is a problem for them with their child, from 1 (not at all) to 7 (very much). On the Confidence scale, parents rate how confident they are in dealing with the problematic behaviors, from 1 (Certain I can’t do it) to 10 (Certain I can do it) [24]. The factor structure was explored in a separate study presenting four factors [104], presented in Table 2. The LBC was developed in Australia and has been validated in the Netherlands [22]. In both countries the instrument has successfully been able to discriminate between healthy weight children and children with overweight/obesity [22, 24] and showed sensitivity to changes after an obesity treatment program [26]. To be able to assess changes in child behavior and parental confidence in the ML we have translated and validated the LBC in a separate study. The LBC was tested in a large Swedish preschool population regarding factor structure, construct validity, internal reliability (Cronbach’s alpha) and discriminative validity [23].

#### Child behavior problems

Child psychosocial health and functioning will be assessed with the Child Behavior Check List for Ages 1.5–5.5 (CBCL/1.5–5.5) that includes 99 questions representing seven syndromes clustering on seven factors. The first four cluster on the Internalizing scale and the last two on the Externalizing scale [105]. The parents rate each behavior from: 0- not true, 1- somewhat or sometimes true and 2- very true or often true. The psychometric properties of the CBCL/1.5–5.5 have been tested for generalizability across 23 countries, including Scandinavian countries. In this large study, including over 19 000 preschool aged children, the CBCL proved to be valid and reliable to measure for emotional, behavioral and social problems in children in different cultures [106]. The CBCL has also been validated in Sweden on a preschool population [107].

#### Parenting practices

Parental feeding practices will be measured with the Child Feeding Questionnaire (CFQ). CFQ consists of 31 items and assesses parents' perceptions and concerns about child obesity, as well as their child-feeding attitudes and practices [108]. The instrument is well suited for use in research concerning parents of preschool-aged children [109]. The CFQ consists of seven factors; the first four factors measure parents’ perceptions of their own and their child’s weight at different ages, and concerns parents may have that can affect how they control their child’s eating; the last three factors measure parental attitudes and feeding practices [108]. In a Swedish validation study the CFQ proved to be a valid and reliable measure in a preschool population after few adjustments [92].

To assess general parenting practices we have developed a questionnaire, Communicating with Children (CC), measuring parents’ limit setting strategies (are they consequent or not) and emotional regulation (are the parents able to control negative emotions when communicating with the child in different situations). Limit setting and emotional regulation are two important aspects discussed and practiced during the parent group sessions. The factor structure, internal reliability, construct validity and discriminative validity will be assessed in a separate study based on the same Swedish preschool population as the one used for validating the LBC, described above.

Parents’ confidence in handling child problematic obesity related behaviors will be measured with the LBC, see above [24].

#### Family function and parental depression

Family functioning will be assessed with the Family Assessment Device (FAD) consisting of 60 items loading on seven factors [110–112]. The instrument has proved to be a reliable tool and can provide valid assessments in a wide range of families [112].

Parental level of depression will be assessed with the Beck Depression Inventory II (BDI-II) [113]. The BDI-II consists of items regarding 21 symptoms and attitudes rated on a 4 point Likert scale from 0 to 3 during the past week. The BDI-II is a commonly used instrument with good psychometric properties proved in many studies on both clinical and non-clinical populations [113].

### Statistical analysis

We will use descriptive statistics (e.g., means, medians, percentages and frequencies) to describe demographic and other subject characteristics and evaluate variable distributions with histograms and boxplots. We will employ several multivariate analytic strategies, including Multi Analysis of Variance (MANOVA), multiple regressions, and structural equation modeling (SEM) controlling for baseline and other covariates. To maximize our multimethod, multiagent approach, we will use the SEM framework most often.

For our analyses we will use SPSS Statistics 23 (IBM, Armonk, NY, USA) and Mplus 7 (Muthen & Muthen. Los Angeles, CA). The advantages of Mplus is that it allows for regression among and between random effects and factors and employs full information maximum likelihood (FIML) estimation to allow for missing data or data that are missing at random (MAR). Even when the MAR assumption is not met, FIML produces less biased estimates than list wise deletion. This feature is particularly advantageous when dealing with longitudinal data.

Latent growth model (LGM) will be used to test intervention effects of the two treatment approaches on decreased BMI SDS. LGM is a preferred method for testing changes in outcome measures in clinical research because it allows for the estimation of average trajectories (mean intercepts and slopes) and individual differences in these trajectories (intercept and slope variances); repeated measures ANOVAs allow for the estimation of mean growth patterns only [114].

**Fig. 3:**
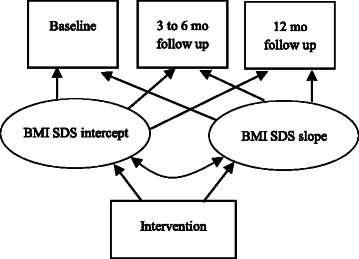
Latent Growth Modeling of children’s BMI SDS

Below we describe specific examples of the analyses that will be conducted to test our aims. In Fig. 3 (Specific Aim 1) we illustrate a LGM where child BMI SDS is modeled as a function of two latent variables, an initial status or intercept factor (representing BMI SDS at baseline) and a slope factor (representing the magnitude of change in BMI SDS over the course of the study). A negative mean for the slope factor in this model would indicate a significant intervention effect on child BMI SDS. For the simplicity of the presentation, the example includes only treatment status as a predictor. However, this model will be expanded to include other hypothesized predictors (e.g., child weight status, parental depression, and SES).

**Fig. 4:**
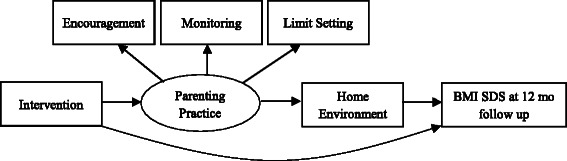
Structural Equation Modeling testing mediating effects of parenting and home environment on BMI SDS

Figure 4 illustrates a model of how the mediating effects of parenting practices and the home environment will be tested with SEM (Specific Aim 3). We expect that increased positive parenting practices in the parent group program will lead to a more positive home environment, which will subsequently lead to decreased BMI SDS. We also expect that the direct effect of the intervention will be reduced in the presence of the two mediators.

**Fig. 5:**
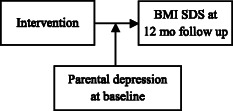
A model testing moderating effects of parental depression

We expect that treatment effect on child BMI SDS will vary depending on socio-economic and demographic factors. Fig.5 illustrates how we will examine the moderating effects of socio-economic and demographic factors using parental depression as an example (Specific Aim 3). We expect that children with parents experiencing higher levels of depression will have a higher BMI SDS at 12 months post-baseline compared to the children whose parents experienced lower levels of depression.

## Discussion

Childhood obesity is one of the greatest challenges facing the modern health care system. Because obesity has proven difficult to treat in adulthood, perhaps a more realistic approach is to start treatment in childhood. Thus, this study aims to fill the current knowledge gap in evidence-based obesity treatment, by carefully evaluating its effectiveness in early childhood (≤ 6 years). Our research results will also add to a broader comprehension and recognition of the family’s role in shaping a child’s lifestyle by determining what parenting practices are most influential early in life and whether these can be modified. This knowledge is important for the development of family-based programs for obesity as well as other non-communicable pediatric diseases such as diabetes and asthma, ultimately improving children’s life-course trajectories and reducing chronic disease risks and associated societal costs.

### Strengths and limitations

#### Target population and recruitment process

To be able to reach a heterogeneous population based sample we have invited all health care representatives in Stockholm County to recruit families to the ML Study. However, there are some factors that might bias the study sample. First, families with parents who do not understand Swedish can unfortunately not be invited to participate in the study for practical reasons (the groups are conducted in Swedish; so far, the group material is only available in Swedish and English; the questionnaires are in Swedish). Secondly, more socioeconomically challenged families are less likely to participate in research studies [115]. The language barrier and the risk of a social economically skewed sample are a concern since obesity rates for preschool aged children in ethnically diverse and socioeconomically challenged areas are high [90, 116]. Thirdly, we are more likely to reach already well-motivated parents who are concerned about their child’s weight. However, we believe the chance of reaching less aware parents will increase by using the primary health care nurse’s skills, knowledge and relationship with the family, even though we are well aware of the challenges the nurse faces when introducing the child’s obesity to the family. We also anticipate that the recruitment of families to the study will be challenging. Previous studies show that parents find it difficult to detect overweight and obesity in their child [117–119]. The parents’ unawareness or reluctance to make the child aware of his or her weight status may raise concerns and be a sensitive task for primary child health care professionals [120]. The everyday stress that most parents experience may be another reason for parents to decline participation in the study with the extensive battery of questionnaires to fill out and, if randomized to the parent group treatment, weekly sessions to attend for 10 consecutive weeks.

#### Sufficient power for detecting secondary outcomes

To our knowledge this is the first longitudinal study with a randomized controlled design evaluating different treatment approaches for childhood obesity in preschool aged children exclusively. One strength of the study is the extensive collection of both measured and self-reported data that enables a thorough examination of key mechanisms for treatment results. However, breaking new ground involves uncertainties and because of the scarcity of previous obesity intervention studies that use the same instruments, the expected changes remain to be seen. Further, even though this is one of the larger studies for this population, we might not have enough power to detect the meaningful changes in our secondary outcomes (the power calculation was based on the primary outcome variable).

#### Variables not included

To measure the secondary outcomes parents are asked to fill out a large battery of questionnaires but instruments for assessing child temperament and parental stress have not been included. Child temperament has previously been reported to interact with parenting [121] and is a behavioral risk factor for child obesity; a more difficult child temperament early in life was associated with higher weight status later in childhood [122–123]. Few studies have focused on parental stress and childhood obesity and show associations to more controlling feeding practices [52]. As we did not want to burden the families with too many questionnaires since this could lead to significant difficulties in data collection [58]; we chose not to include measures of child temperament and parental stress. However, we do measure child behavior with the CBCL/1.5–5. That we use this version of the CBCL could be questioned since our population includes 6-year-old children; but because children in Sweden start school at the age of seven the CBCL/1.5–5 seems to be a better fit for our sample of preschoolers.

The scarcity of studies evaluating parenting and its role in obesity can be explained in part by the lack of validated questionnaires [58]. In two separate studies we will validate the Australian developed instrument, the LBC [24] and a self-developed questionnaire, the CC. The LBC has been translated and the psychometric properties have been examined including the factor structure [23]. The development of the Swedish version was conducted in collaboration with the Australian founders. CC examines what limit setting strategies parents use with their child, if they are being consequent or not, and parents ability to emotional control. The questionnaire was developed and its psychometric properties will be assessed according to international guidelines examining: face and content validity, factor structure, internal reliability, construct and discriminative validity [125, 126]. The focus on parenting in ML makes the two instruments valuable for the evaluation of the study.

#### Self-reported data

The data collected for the evaluation of ML is to a large extent self-reported. Using self-reported data we need to be careful when interpreting the results because of the risk for bias. In a study by Farrow and colleagues mothers underreported controlling child feeding practices compared to the parenting practices observed in a laboratory setting [127]. The authors discussed whether this was due to social desirability or whether parents are simply unaware of their behavior. Considering the latter, communicating alternative strategies for parents to use in feeding situations with their obese child may be important to address in the obesity treatment.

#### Lack of blinding

It should be noted that the same research staff is involved in measurements of the children randomized to the parent group treatment, conducting the parent groups and performing the booster session calls. This setup introduces a risk for bias and a more objective execution and examination would have been preferred. However, the research staff is well educated and have years of experience working with families with obese children both in clinical and research settings and we believe this may minimize the risk of bias.

#### Lack of non-treated control group

A final limitation in the study is the lack of a non-treated control group which would have strengthened the study design further. In the planning stages of the study we decided against an un-treated control group due to ethical considerations; it is not ethical to withhold treatment from children with a chronic disease if there is a treatment to offer [18]. That being said, a common critique to obesity treatment targeting preschoolers is the possibility that we are treating children who would have grown out of the obesity without treatment; especially children less genetically susceptible to obesity, as shown by Whitaker and colleagues [128]. Although the cost-effectiveness of treatment for children who could become normal-weight on their own can be questioned, treatment offered to participating families in this study is based on lifestyle and parenting advice – information that is helpful for all parents regardless of the child’s weight. Additionally, it is well documented that the incidence of obesity increases with age [129].

## Conclusions

The urgent need for decreasing the prevalence of childhood obesity makes research on developing programs that can be adapted into clinical practice highly relevant. The ML Study has been developed and is performed in close collaboration with stakeholders within the primary and secondary child health care systems in Stockholm County. This design will enable us to investigate the feasibility of the intervention in already existing systems and also to simplify the scale up of the intervention if proven to be effective. Further, the study will be able to demonstrate the clinical effectiveness of different treatment approaches for childhood obesity, specifically: the optimal focus (parenting practices or lifestyle changes), optimal treatment length and intensity and other key determinants influencing treatment outcomes (e.g., SES, parental depression). In summary, this study will bolster the limited evidence base in this field and provide results highly relevant to the design of future early childhood obesity treatment programs.
